# Evaluating the Usefulness of Artificial Intelligence-based Chest X-Ray Screening in Improving Tuberculosis Detection Among the High-Risk Tribal Population of Chhattisgarh, India: A Prospective Multi-Centre Study

**DOI:** 10.1093/ofid/ofaf780

**Published:** 2026-01-07

**Authors:** Abhishek Gupta, Aswathy M Nair, Shobha Ekka, Dharmendra Gahwai, Nisha Sharma, Faisal Raza Khan, Manisha Damani, Suraj Kumar, Saniya Pawar, Justy Antony Chiramal, Dennis Robert, Manoj Tadepalli, Shibu Vijayan, Pranav S. Krishnan, Nidhi A. Patil

**Affiliations:** Tribal TB Initiative, Piramal Swasthya Management and Research Institute, Chhattisgarh, India; Qure.ai Technologies Pvt Ltd, Clinical, R&D, and Operational Research Division, Bengaluru, India; Tribal TB Initiative, Piramal Swasthya Management and Research Institute, Chhattisgarh, India; State TB Officer, Department of Health & Family Welfare, Chhattisgarh, India; Tribal TB Initiative, Piramal Swasthya Management and Research Institute, Chhattisgarh, India; Tribal TB Initiative, Piramal Swasthya Management and Research Institute, Chhattisgarh, India; National TB Elimination Programme, National Health Mission, Government of Chhattisgarh, Chhattisgarh, India; Tribal TB Initiative, Piramal Swasthya Management and Research Institute, Chhattisgarh, India; Qure.ai Technologies Pvt Ltd, Clinical, R&D, and Operational Research Division, Bengaluru, India; Qure.ai Technologies Inc., Product Division, New York City, New York, USA (Remote Work from Smyrna, Georgia, USA); Qure.ai Technologies Pvt Ltd, Clinical, R&D, and Operational Research Division, Bengaluru, India; Qure.ai Technologies Pvt Ltd, Clinical, R&D, and Operational Research Division, Bengaluru, India; Qure.ai Technologies Pvt Ltd, Clinical, R&D, and Operational Research Division, Bengaluru, India; Qure.ai Technologies Pvt Ltd, Clinical, R&D, and Operational Research Division, Bengaluru, India; Qure.ai Technologies Pvt Ltd, Clinical, R&D, and Operational Research Division, Bengaluru, India

**Keywords:** analog films, artificial intelligence, chest x-ray, tribal, tuberculosis

## Abstract

**Background:**

India accounts for the highest Tuberculosis (TB) burden globally. The incidence and prevalence of TB are higher in tribal population than general population. In this study, we assessed the effectiveness of artificial intelligence (AI) based chest X-ray (CXR) interpretation software device (qXR version 3), in detecting TB from a predominantly tribal population setting.

**Methods:**

In this multicenter prospective study, all the CXRs of patients aged > 15 years taken for any reason at 3 public health facilities in the Chhattisgarh state of India between 01 August 2023 and 31 March 2024 were included. Patients flagged by AI as TB presumptive were directed to undergo sputum testing, who are subsequently confirmed either microbiologically or clinically.

**Results:**

Out of 2745 CXRs screened, 363 patients (median age, 44 years [IQR: 30–53]; 261 [71.9%] male) were identified as presumptive for TB. 162 cases were confirmed with TB positivity rate of 44.63% (95% CI: 39.44–49.91). Among the AI-flagged cases, 51 (14.04%) patients were asymptomatic, and 20 (39.22%) of them were confirmed with TB. Descriptively, when compared with baseline (August-2022 to March-2023), an 80.21% (*P* < .001) increase in the number of TB case notifications was observed during the AI implemented period.

**Conclusions:**

This study highlights the potential of AI to enhance TB detection and feasibility in a resource-limited tribal setting. Above 40% of the patients flagged by AI were subsequently confirmed to have the TB disease. Additionally, the study demonstrated the potential of AI in identifying asymptomatic individuals who would otherwise have been missed or diagnosed late.

## Teaser

This article presents the findings of a multicenter, prospective study that evaluated the effectiveness of AI software for analog chest X-ray interpretation in identifying tuberculosis (TB) among high-risk tribal population. The AI-enabled workflow have supplemented symptomatic screening, enhancing TB detection at the facilities with high positivity rate. Notably, the AI also identified asymptomatic patients who would have been missed otherwise in typical symptom-based TB screening approach.

## Graphical Abstract

**Graphical Abstract:**
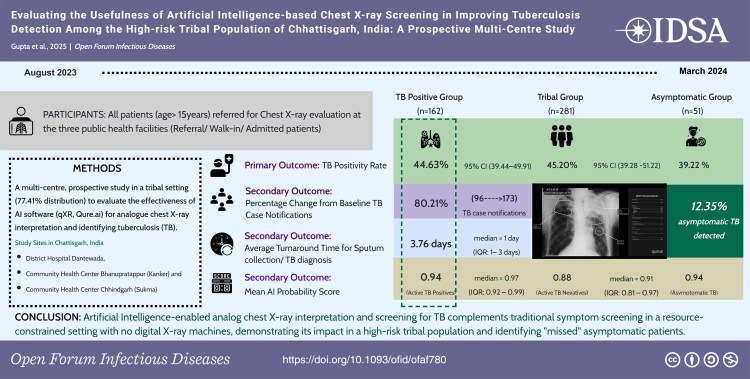
This graphical abstract is also available at Tidbit: https://tidbitapp.io/tidbits/evaluating-the-usefulness-of-artificial-intelligence-based-chest-x-ray-screening-in-improving-tuberculosis-detection-among-the-high-risk-tribal-population-of-chhattisgarh-india-a-prospective-multi-centre-study?utm_campaign=tidbitlinkshare&amp;utm_source=ITP

Tuberculosis (TB) is one of the leading causes of death and a public health concern on a global scale [1]. An estimated 10.8 million individuals contracted TB in the year 2023, as per the World Health Organization (WHO) report, claiming about 1.25 million lives. India accounts for the highest burden of TB globally, with about 27% (195 incidences per 100 000 population) of the patients, as well as contributing to a total of 315 000 deaths due to TB [2]. The national strategic plan (NSP) for TB elimination targets to reduce the incidence of new patients with TB in India to 44 per 100 000 population by the year 2025, but this seems to be difficult to achieve given the current burden of disease [3]. Missed diagnoses significantly contribute to the spread and burden of the disease, and the number of “missed” patients of TB remains high, estimated at 3.9 million globally and about 600 000 in India alone [1]. An untreated active patient with TB can infect an average of 10–15 people per year [4].

The India TB Report 2023 showed a 13% increase in TB notifications in 2022 compared with 2021, largely due to sustained active and passive case finding, enhanced private sector engagement, and improved presumptive TB examination rates leading to the identification of previously undetected patients [5]. However, sub-nationally, the impact of TB burden and epidemiology differs in both urban and rural settings. While the urban areas face a higher transmission rate of disease due to population density, the rural areas exhibit higher prevalence and longer recovery times because of limited access and awareness [6], thereby requiring specific yet interconnected interventions to control the disease. Additionally, lack of adequate resources, regulated care delivery, and limited infrastructure make TB control in rural settings challenging [7].

As per the Tribal TB initiative of 2021 [8], the tribal zones account for about 10.4% of total TB notifications in India. The incidence and prevalence of TB in the tribal population in India are both known to be 2–3 times more than the national averages [9]. This highlights the need for targeted efforts to actively identify patients with TB and implement comprehensive interventions to reduce the disease burden in high-risk tribal populations [10]. In India, Chhattisgarh is one of the states with the highest disease burden and disability-adjusted life years rate for TB. The state has also consistently recorded higher TB notifications in the past years [11].

Chest X-ray (CXR) is a crucial imaging tool for pulmonary TB screening and diagnosis [12]. The National TB Elimination Programme (NTEP) recommends an integrated diagnostic approach emphasizing the use of CXR as a triaging tool for TB, followed by bacteriological confirmation through molecular WHO-recommended rapid diagnosis like Cartridge-Based Nucleic Acid Amplification Tests (CBNAAT) [13]. In resource-constrained remote settings, limited access to trained clinicians and resources may lead to delays in reporting CXRs and diagnosis, potentially impacting the care cascade. Furthermore, addressing the subjective inter- and intra-reader variability in interpreting CXRs is also critical [14–16].

Evidence suggests that artificial intelligence (AI) algorithms have the potential in tackling TB burden by mitigating some of these challenges. WHO has advocated the use of computer aided detection products with AI as an alternative to human interpretation for screening pulmonary TB in adults using CXRs [17, 18]. In addition to digital CXR interpretation, AI tools can also assist with stratifying risk scores, indicating the likelihood of TB and allocating resources for further evaluation accordingly [3, 19]. For implementing site-specific interventions in resource-constrained tribal settings, more data and evidence [18, 20] about the clinical effectiveness of AI for TB detection are required. In this study, we assessed the usefulness of a validated AI-based CXR interpretation software (qXR, Qure.ai) device in identifying presumptive patients with TB from conventional plain film CXRs in a setting where the population is predominantly tribal.

## METHODS

As this study was done as a part of service delivery evaluation of an already regulatory cleared device, a waiver for the requirement of informed consent was obtained and the study was initiated in agreement with the State TB Officer. Ethics committee approval was obtained for conducting this research.

### Study Design and Population

This was a multicenter, prospective study, and it included individuals who underwent CXR examination for any reason at 3 public health facilities in 3 districts (District Hospital in the Dantewada district, Community Health Center Bhanupratappur in the Kanker district, and Community Health Center Chhindgarh in the Sukma district) in Chhattisgarh state in India during a period of 8 months from 01 August 2023 to 31 March 2024. The average daily CXR volume processed at 1 facility ranges from 10 to 15 scans, while at the others, it is between 2 and 5 scans. The study setting was selected due to its disease prevalence, presence of tribal population, operational feasibility, availability of X-ray systems, highest vulnerability due to limited private facilities or resources and remoteness. None of the sites had digital X-ray infrastructure, and they relied on conventional plain film radiographs. The percentage of the tribal population in these districts is reported to range from 55% to 77% [21].

Patients aged ≥ 15 years who have undergone a posteroanterior/anteroposterior CXR for any reasons at the study site with an AI score more than or equal to the preset threshold of 0.65 were considered eligible for the analysis. A threshold AI score of 0.65 was chosen to balance sensitivity and specificity for TB detection, based on prior implementation learnings. This score helps identify true positives at an acceptable false positive rate. The patients below this threshold will follow the standard TB screening process. Patients with already diagnosed TB who were taking CXR as part of follow-up, and those with a lack of TB confirmation results, were excluded. Along with patient age, gender, and clinical symptoms, the demographic data of the individuals, including their tribe and sub-tribe, were also captured for analysis. Only the patients with symptoms suggestive of TB, like chest pain, weight loss, fever, persistent cough, body ache/pain, difficulty in breathing, fatigue, night sweats, and/or chills were considered to be symptomatic, and the others as asymptomatic.

### Study Workflow

**Figure 1.**
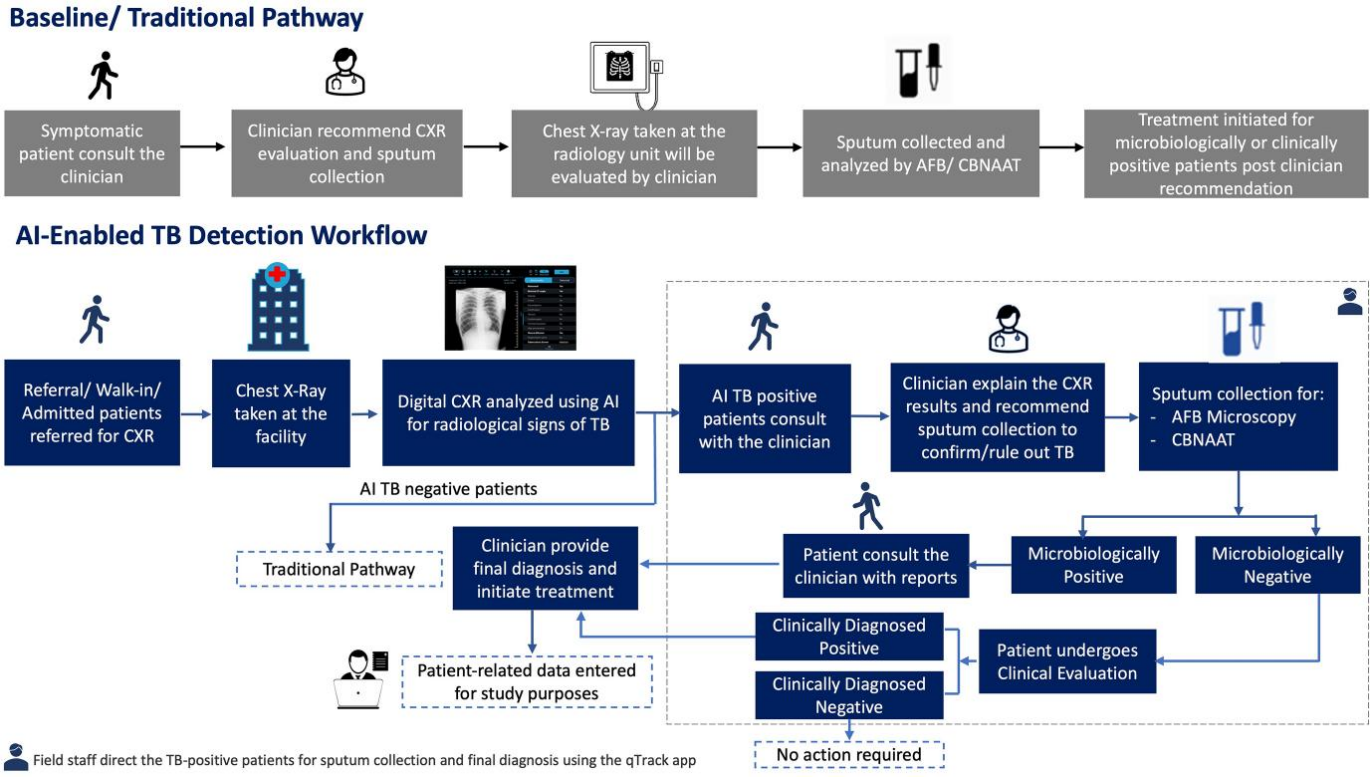
Study workflow followed at the data collection sites with and without AI deployment. Here the AI-enabled TB detection workflow supplements the current standard of care based on symptomatic screening.

The baseline TB pathway followed at the site before AI deployment involved initial screening for clinical symptoms of TB, and if the patient is clinically symptomatic, further CXR evaluation and confirmatory TB testing are performed. In the AI-enabled TB screening workflow (Figure 1), all individuals undergoing CXR evaluation at the study site (also including referral CXRs taken for reasons not related to TB) were screened by AI. One trained field coordinator was deputed in each of the 3 facilities. They manually uploaded the photos (jpeg/png format) of the analog CXR films taken against a view box (process instructions for image capture are given in S4, and training material shared as supplementary information) using regular smartphones into the software (qTrack version 3, Qure.ai) for processing. qTrack is an end-to-end disease management platform that is used to upload a CXR image, to view the images and AI results, to enter patient details, diagnostic test results, and connect healthcare workers and patients.

If the CXR was flagged positive for TB signs by AI (indicated by an AI probability score ≥ 0.65), those patients were directed to a sputum test for AFB smear microscopy and CBNAAT (GeneXpert) testing to confirm the TB diagnosis on microbiological grounds. Among those who had negative microbiological tests, some patients were clinically diagnosed as TB by the clinician based on their symptoms and signs. The treatment was then initiated for the TB-confirmed patients as per the site guidelines or the clinician's recommendation.

### Statistical Analysis

The primary objective of this study was to determine the TB positivity rate among patients deemed presumptive for TB by the AI. The TB positivity rate was defined as the proportion of AI-flagged patients subsequently confirmed as TB positive. Subgroup analysis for TB positivity rate stratified by age, gender, tribe, and whether or not the patient was symptomatic are also reported. A composite reference standard (bacteriological or clinical diagnosis of TB) was used for TB diagnosis.

As secondary analyses, we report the percentage change in TB notifications during the AI implementation period (August 2023 to March 2024) with that of a baseline period (August 2022 to March 2023). The baseline period had no AI at the sites and was chosen to have similar months as that of AI implementation period to mitigate seasonal confounding of TB incidence. Only aggregate data was available for the baseline period from historical records and only descriptive comparison is reported. Summary statistics of turnaround time in days from CXR acquisition date to sputum collection date and to TB diagnosis date are also reported.

We also investigated the AI probability score distribution and its characteristics in important subgroups. Welch's *t*-test was performed to assess if the mean of the scores is statistically different in true positive versus false positives, in asymptomatic versus symptomatic TB positive patients and in clinical versus microbiologically diagnosed patients.

### Artificial Intelligence Software Device Description

qXR (version 3), a Software as a Medical Device, developed by Qure.ai, is an AI-driven CXR interpretation software used in this study (more information is given in S1, supplementary information). The diagnostic accuracy of the AI algorithm has been validated for TB detection through multiple studies globally, demonstrating 0.93 sensitivity and 0.75 specificity for culture-confirmed TB [22]. In a high TB burden setting of Bangladesh, the algorithm has reported an AUC (area under the receiver operating characteristic curve) of 90.81% outperforming human readers, fulfilling the WHO's target product profile criteria (minimum 70% specificity at 90% sensitivity) and reducing the requirement of GeneXpert by 50% [16]. Similarly, in India, an active case finding study has demonstrated an additional 15.8% increase in the diagnostic yield of TB confirmed patients that are identified only due to the AI integration in the conventional care cascade [21, 23]. Involving AI for mass screening in high-risk prison settings has also proven effective with improved disease detection and acceptable sensitivity [22, 24]. AI works with both digital (DICOM CXR files) and analog (eg, photos of CXR films captured using regular smartphones) CXRs [16, 23] as inputs and is regulatory cleared for screening and triage of pulmonary patients with TB for routine clinical use.

## RESULTS

### Baseline Characteristics

**Figure 2.**
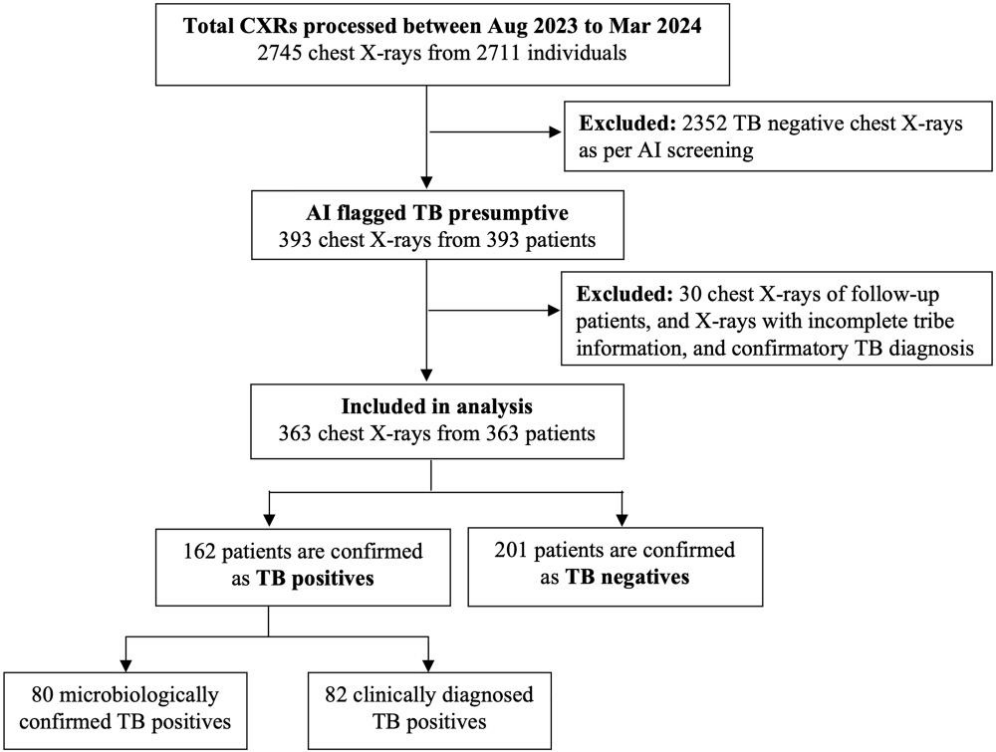
Study data flow diagram.

Out of 2745 CXRs processed during the study period, 393 CXRs from 393 distinct patients were flagged as presumptive for TB by the AI software. However, only a total of 363 patients were included in the final analysis, as explained in Figure 2. In addition, 17 of the 363 patients did not undergo sputum testing due to patients not being able to produce sputum or refusing to test.

The baseline characteristics of the 363 patients included in the analysis are shown in Table 1. The median age of the selected population was 44 (IQR: 30–53), and 71.90% were male. 14.04% of asymptomatic patients were flagged by AI as TB presumptive, leading to their inclusion in the care pathway. Of the 281 (77.41%) patients from the tribal communities, 248 (68.31%) were from the Gond tribe. The other comparatively smaller sub-categories of tribal groups that are represented in this study include Halba (7.11%), Dhurwa (2.49%), Muriya (1.77%), and Kunwar (0.35%).

**Table 1.** Baseline Characteristics

Characteristic	Overall	TB Positive Group	TB Negative Group
	n = 363	n = 162	n = 201
Gender			
Male, n (%)	261 (71.90)	121 (74.69)	140 (69.65)
Female, n (%)	102 (28.09)	41 (25.31)	61 (30.35)
Age (in y)			
Mean + SD	42.88 ± 15.68	42.85 ± 15.69	42.88 ± 15.68
Median (IQR)	44 (30–53)	43.50 (30–53)	44 (30–53)
Age group (in y)			
16–30, n (%)	98 (26.99)	54 (33.33)	44 (21.89)
31–65, n (%)	236 (65.01)	103 (63.58)	133 (66.17)
> 65, n (%)	29 (7.98)	5 (3.09)	24 (11.94)
Clinical symptoms			
Symptomatic, n (%)	312 (85.95)	142 (87.65)	170 (84.58)
Asymptomatic, n (%)	51 (14.04)	20 (12.35)	31 (15.42)
Tribe			
Tribal, n (%)	281 (77.41)	127 (78.40)	154 (76.62)
Non-Tribal, n (%)	82 (22.58)	35 (21.60)	47 (23.38)
Site			
District Hospital Dantewada, n (%)	240 (66.12)	96 (59.26)	144 (71.64)
Community Health Center Bhanupratappur, n (%)	60 (16.53)	52 (32.10)	8 (3.98)
Community Health Center Chhindgarh, n (%)	63 (17.35)	14 (8.64)	49 (24.38)

Abbreviations: N: Absolute number of patients;

SD, standard deviation;

IQR, inter-quartile range.

### Primary Analysis: Tuberculosis Positivity Rate

Of the 363 presumptive patients with TB, 162 were diagnosed to have TB based on either microbiological (n = 80) or clinical (n = 82) grounds. Among the 80 microbiologically diagnosed patients, 32 (40.00%, 95% CI: 29.20–51.56) were confirmed using AFB smear microscopy and 48 (60.00%, 95% CI: 48.44–70.80) were confirmed using CBNAAT/GeneXpert. This translates to a TB positivity rate of 44.63% (95% CI: 39.44–49.91) in patients flagged by AI with the presence of radiological signs of TB (Table 2). Among the 80 microbiologically detected patients, 32 (40.00%, 95% CI: 29.20–51.56) are confirmed using AFB smear microscopy and 48 (60.00%, 95% CI: 48.44–70.80) are confirmed using CBNAAT/GeneXpert. The microbiological test positivity rate was found to be 23.12% (95% CI: 18.78–27.93). It is also important to note that 12.35% of the 162 TB confirmed patients were asymptomatic with no notable symptoms of TB.

**Table 2.** TB Positivity Rate

Group	TB Positivity Rate In Percentage (95% CI)
	Median (IQR)
Overall (n = 363)	44.63 (39.44–49.91
Age in years	
16–30 (n = 98)	55.10 (44.72–65.17)
31–65 (n = 236)	43.64 (37.22–50.23)
> 65 (n = 29)	17.24 (5.85–35.77)
Gender	
Male (n = 261)	46.36 (40.19–52.61)
Female (n = 102)	40.20 (30.61–50.37)
Symptoms	…
Symptomatic (n = 312)	45.51 (39.89–51.22)
Asymptomatic (n = 51)	39.22 (25.84–53.89)
Population	
Tribal (n = 281)	45.20 (39.28 -51.22)
Non-tribal (n = 82)	42.68 (31.82–54.10)
Site/ Facility	
District Hospital Dantewada	40.00 (51.27–66.90)
Community Health Center Bhanupratappur	86.67 (75.41–94.06)
Community Health Center Chhindgarh	22.22 (12.72–34.46)

Further, TB positivity rate stratified by age group, gender, symptoms and tribal population is shown in Table 2. In males, the TB positivity rate was found to be comparatively higher (46.36, 95% CI: 40.19–52.61) than in females (40.20, 95% CI: 30.61–50.37), whereas it was found to be lower (17.24, 95% CI: 5.85–35.77) in the elderly population aged > 65 years. The TB positivity rate in the asymptomatic population (39.22%, 95% CI: 25.84%–53.89%) was observed to be lower than that in the symptomatic population (45.51%, 95% CI: 39.89–51.22). The TB positivity rates are also found to vary among the tribal (45.20, 95% CI: 39.28–51.22) and nontribal (42.68, 95% CI: 31.82–54.10) subgroups.

### Secondary Analyses

#### Change in the Number of Tuberculosis Notifications With and Without Artificial Intelligence

During the baseline (August–March period of 2022–23), there were 96 patients with TB from the 3 selected sites. In this AI implementation study, which was conducted during August–March of 2023–24, we found that 173 patients with TB were notified with TB confirmation. This indicated an 80.21% percentage increase in the number of TB notifications during the period when AI was active in the sites compared with the baseline period. It is to be noted that this is only a descriptive analysis, and further research by collecting more time series data with analysis incorporating adjustments for potential confounders is needed to conclude if the increase in TB notifications can be attributed to AI.

#### Turnaround Times

The average time difference between the CXR acquisition date and TB diagnosis date was found to be 4 days (median: 2 days, IQR: 1–4 days), and the average time difference between the CXR acquisition date and sputum collection date was found to be 3.76 days (Median: 1 day, IQR: 1–3 days).

#### Artificial Intelligence Probability Score Analysis

Higher TB positivity rates were observed in the strata of higher scores (S3 in supplementary depicts all the TB positivity rates for different ranges of AI probability scores) of the patients whose CXR had an AI probability score in the range of 0.91–1.00 were diagnosed with TB (TB positivity rate: 54.59, 95% CI: 47.89–61.16).

About 75% of the AI scores were ≥ 0.86 (median: 0.94, IQR: 0.86–0.98) in the overall sample (Table 3). In patients who were diagnosed with TB, about 75% of AI scores were ≥ 0.92 (median: 0.97, IQR: 0.92–0.99). The mean AI probability scores in TB positive (true positive) patients (0.94) were significantly larger than that in TB negative (false positive) patients (0.88, *P* < .001) indicating that there is higher likelihood that a patient with a higher AI score for his/her CXR will turn out to be a true positive patient than false positive (S2 of supplementary information). Within the TB positives (n = 162), the AI probability scores were higher in microbiologically confirmed patients (0.96) than in clinically diagnosed ones (0.92, *P* = .002). The mean probability score in symptomatic and asymptomatic patients was 0.94 each (*P* = .80), indicating that asymptomatic patients who were finally diagnosed with TB had comparable AI scores with those of symptomatic patients even in the absence of TB-specific symptoms.

**Table 3.** Summary Statistics for Distribution of AI Probability Scores

…	Overall	Active TB Positive Group (True Positives)		Active TB Negative Group (False Positives)
	n = 363	n = 162		n = 201
Mean ± SD	0.91 ± 0.09	0.94 ± 0.08		0.88 ± 0.09
Median (IQR)	0.94 (0.86–0.98)	0.97 (0.92–0.99)		0.91 (0.81–0.97)
…	−^a^	**Microbiologically confirmed patients (n** **=** **80)**	**Clinically diagnosed patients (n** **=** **82)**	-
Mean ± SD	-	0.96 ± 0.07	0.92 ± 0.08	-
Median (IQR)	-	0.98 (0.96–0.99)	0.95 (0.89–0.98)	-
…	-	**Symptomatic patients (n** **=** **142)**	**Asymptomatic patients (n** **=** **20)**	-
Mean ± SD	-	0.94 ± 0.08	0.94 ± 0.07	-
Median (IQR)	-	0.97 (0.92–0.99)	0.97 (0.92–0.99)	-

Abbreviations: SD, standard deviation;

IQR, inter-quartile range.

^a^Not applicable.

## DISCUSSION

In this prospective study, 2745 CXRs were processed by the AI software from the TB prevalent sites and 363 CXRs from 363 patients were flagged as TB presumptive by AI and included for the analysis. The TB positivity rate in 363 patients was found to be 44.63% (95% CI: 39.44–49.91), which is higher than other reported studies in the literature (25). Moreover, the reason for this observed difference with prior reported TB positivity rates is likely due to differences in the study population. Our study was conducted in a rather high-prevalence TB setting, and therefore, the pretest probability for TB disease can be assumed to be higher for this population. Among the 3 sites selected for this study, one of the community health centers showed a higher TB positivity rate of 86.67% (95% CI: 75.41–94.06), and it is observed that in this facility, 85% of the confirmed patients with TB were clinically diagnosed. Nearly half of the patients deemed presumptive for TB as per AI were found to be true positives, indicating good TB positivity rates, especially in those with higher AI probability scores. We also noticed an 80.21% increase in the TB notifications at the study sites while comparing with and without AI deployment phases; however, further research is needed to conclude if the observed increase in TB notifications can be attributed to AI.

The distribution of AI probability scores in asymptomatic (mean: 0.94, SD: 0.07) and symptomatic (mean: 0.94, SD: 0.08) patients also did not show any significant difference (*P* value-.80). We also observed that the higher the AI probability score, the higher the chance that the patient will be likely diagnosed with TB, which corroborates findings from other literature [25]. Interestingly, 51 (14.04%) of the 363 patients were asymptomatic, and 20 of these patients were finally diagnosed with TB. Asymptomatic patients are more likely to end up being missed or have a delayed diagnosis. The National Prevalence Survey of India also support the study findings by directing 42.6% of asymptomatic TB individuals into the care pathway through CXR analysis, who would have possibly remained undetected otherwise [26]. We observed a higher TB positivity rate in the younger population (16–30 years of age). This group is relatively more likely to be socially more active and thus can result in higher transmission of infection to others, and are also known to have substantial loss to follow-up, low treatment adherence, possible drug resistance, disease progression, and mortality risk [27].

In alignment with the NTEP (Detect, Treat, Prevent, and Build) strategy, early detection of presumptive patients with TB, appropriate linkage to testing or care, intensified screening efficiency of health facilities, and vulnerability mapping of high-risk populations are needed to strengthen case detection and management [3, 11]. From the study findings, it is clear that the AI integration complements these targeted NTEP/NSP goals by rapid triaging of CXRs with higher disease likelihood, enabling confirmatory testing and diagnosis without delay, and identifying asymptomatic individuals to reduce the TB incidence. Our study also demonstrates that AI can be deployed in resource-constrained settings lacking digital CXR infrastructure by integrating with conventional analog X-ray systems. Prior studies of the same AI device have shown comparable performance while processing both analog and digital CXR images, emphasizing its adaptability and robustness in multiple systems [14]. Further, the other operational challenges qualitatively pointed by the hospital staff include shortage of resources, geographical constraints, limited diagnostic supplies and long wait times for confirmatory test reporting, loss to follow-up, etc. which further support the adoption and use of AI in such settings.

The main strength of our study is that we demonstrated a high TB positivity rate and yield, enabling improved TB detection in a resource-constrained tribal setting (over 77% assessed were from the tribal population). Thus, by strengthening the diagnostic efforts of the National TB program in the tribal settings, the digital innovations have the potential to reduce the TB burden in prevalent states of India. The limitation of the study is that a comprehensive comparison of the effectiveness of AI with and without AI was not possible due to the lack of robust baseline data, limiting the analysis of change in TB notifications to a descriptive level. Although 2745 CXRs were processed by AI during the study period, we included only the patients whose CXRs were flagged as presumptive for TB by the AI. Therefore, the sensitivity and specificity of the AI could not be determined from our data. Our purpose was to mainly study the TB positivity rates and the usefulness/feasibility of AI in a tribal population and in sites where only conventional plain film radiographs are available. The unavailability of analog CXR films at the facilities during certain time periods has also impacted the overall sample size.

## CONCLUSION

This study underscores the potential of including the AI-enabled CXR-based TB detection pathway in addition to conventional symptom screening for improved TB positivity rate. The integration of AI software into the clinical workflow was also successful in a remote setting with no digital X-ray machines, demonstrating impact in a high-risk tribal population. We also found that AI can help identify asymptomatic patients who otherwise could have likely ended up as “missed” or as late diagnosed patients with TB.

## Supplementary Material

ofaf780_Supplementary_Data

## Supplementary Data

Supplementary materials are available at *Open Forum Infectious Diseases* online. Consisting of data provided by the authors to benefit the reader, the posted materials are not copyedited and are the sole responsibility of the authors, so questions or comments should be addressed to the corresponding author.
