# Supplementary material for: Evaluating the Usefulness of Artificial Intelligence-based Chest X-Ray Screening in Improving Tuberculosis Detection Among the High-Risk Tribal Population of Chhattisgarh, India: A Prospective Multi-Centre Study
Source: Open Forum Infect Dis. 2026 Jan 7;13(1):ofaf780. doi: 10.1093/ofid/ofaf780 (PMC12810203; doi:10.1093/ofid/ofaf780)
Supplement: ofaf780_Supplementary_Data [file ofaf780_supplementary_data.zip › Supplementary Information_Revised (1).docx]

**Supplementary Information**

**S1: An example of an AI-generated image showing the radiological signs of TB as detected by the TB detection model in qXR.**


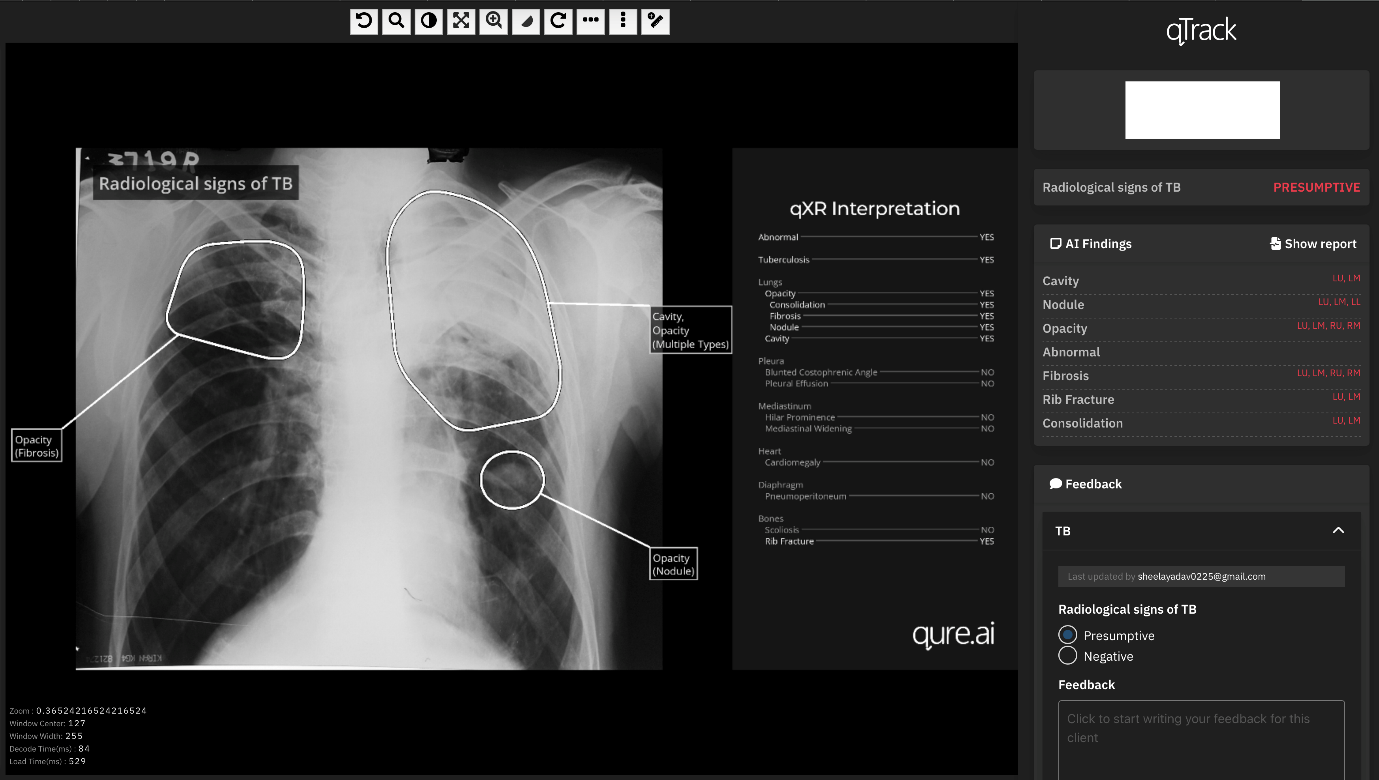


Trained on a data library of about 4.2 million CXR images, the TB detection model used in this study, AI (qXR) detects the presence or absence of the radiological signs of TB. The model generates a numerical probability score between 0 and 1, indicating the predicted probability for the presence of radiological signs of TB. A pre-defined threshold (0.65) is applied to the probability score to classify a CXR for the presence or absence of radiological signs of TB. This threshold of 0.65 for the AI probability score was slightly higher than the most commonly used threshold of 0.5. While a lower threshold might have resulted in a different TB positivity rate, the overall increase in TB notifications during the study period suggests that the chosen threshold was effective in helping to increase case-finding compared to the baseline.

**S2. Distribution of AI probability scores stratified by clinical or microbiological confirmation and presence or absence of symptoms**


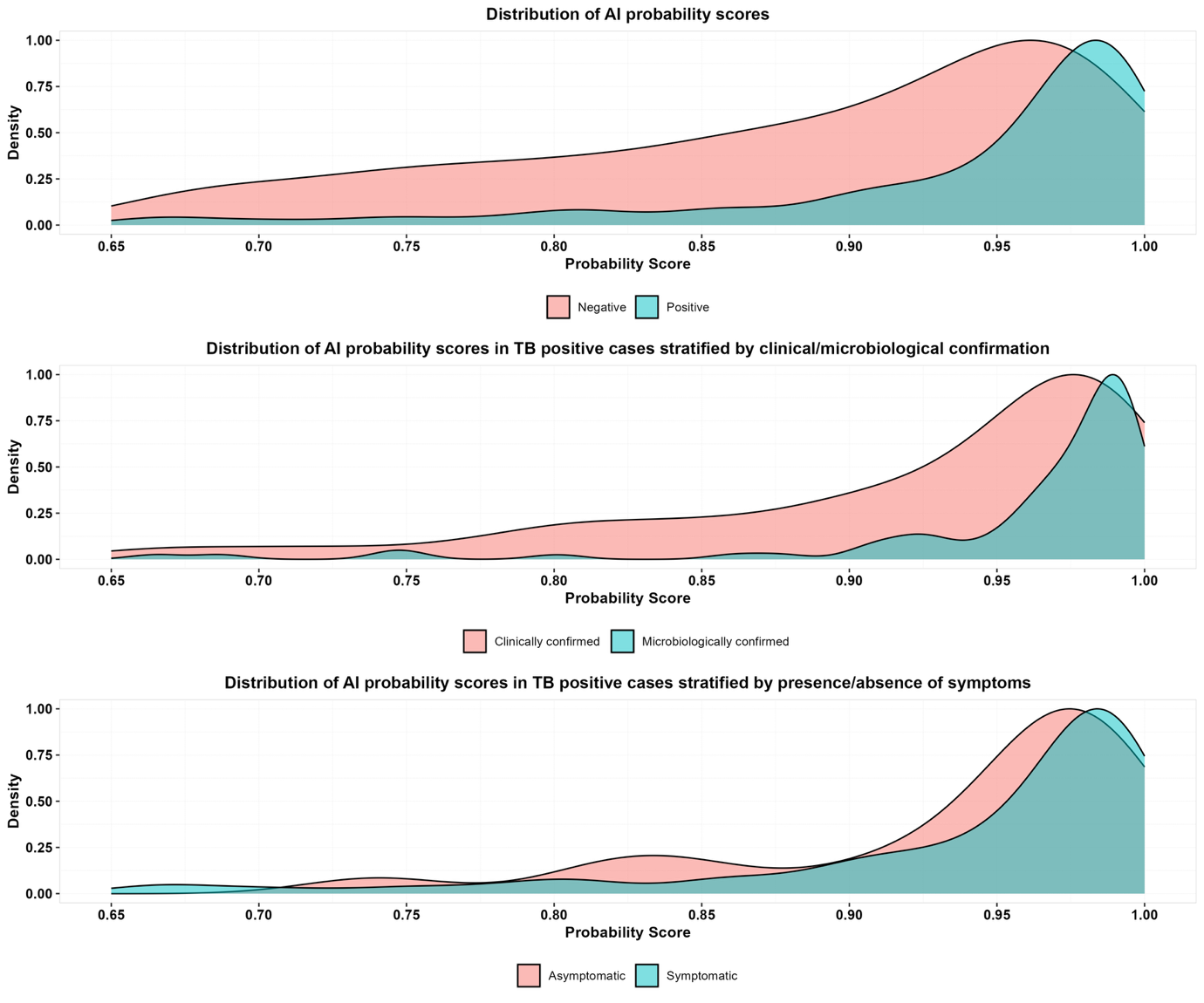


**S3: TB positivity rates in different strata of AI probability score**

| **AI Probability Score** | **Number of patients**  **(N=363)** | **Number of patients confirmed with TB (N=162)** | **TB Positivity Rate**  **(95% CI)** |
| --- | --- | --- | --- |
| 0.65-0.70 | 19 (5.23%) | 5 (3.08%) | 26.32 (9.15-51.20) |
| 0.71-0.80 | 43 (11.84%) | 10 (6.17%) | 23.26 (11.76-38.63) |
| 0.81-0.90 | 72 (19.83) | 22 (13.58%) | 30.56 (20.24-42.53) |
| 0.91-1.00 | 229 (63.08%) | 125 (77.16%) | 54.59 (47.89-61.16) |

This study included patients who are flagged by AI as a presumptive TB case by AI and patients with clinical symptoms from the conventional pathway as well. A patient confirmed to have a diagnosis of TB based on either microbiological or clinical grounds was considered to be a positive case and if the diagnosis of TB was negative based on both microbiological and/ or clinical investigations, then it was considered as a negative case.

**S4: Guidelines and instructions to capture digital images of analogue chest X-ray films**

A simple photo of the conventional plain film CXR captured using regular smartphones guided by the following simple-to-follow instructions can enable AI-assisted TB detection in settings without digital CXR infrastructure.

| 1 | **Setting:** Before clicking the picture, the analogue chest X-ray film should be clipped to a light box that is switched ON | 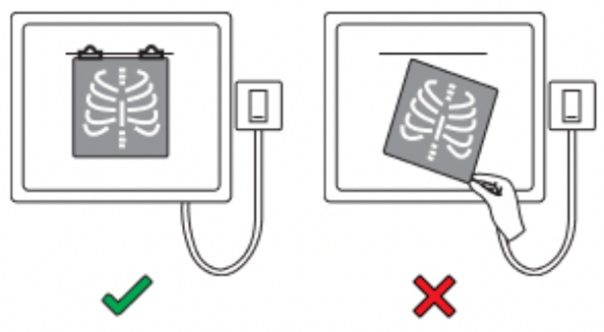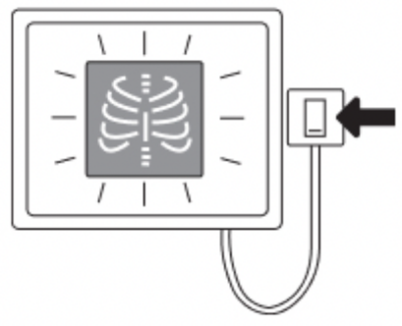  Light box switched “ON” |
| --- | --- | --- |
| 2 | **Background**: The pictures should be taken only using a light box. | 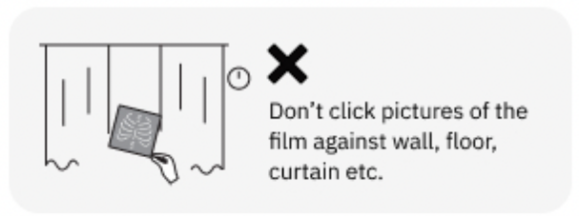 |
| 3 | **Picture quality:** For optimal picture quality, the room should be darkened to minimize ambient light reflections or noises. | 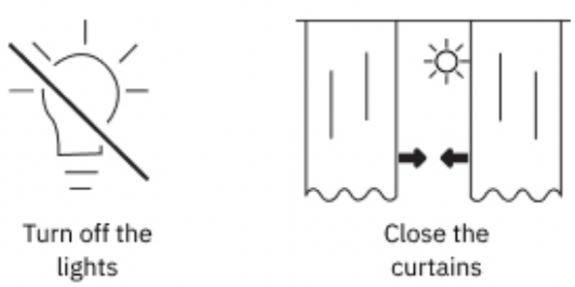 |
| 4 | **Distance**: It is ideal to maintain a 2ft distance between the light box and mobile phone camera. | 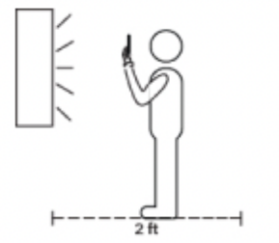 |
| 5 | **Device parameters**: The mobile phone flashlight should be turned OFF while capturing an image to avoid light reflections. | 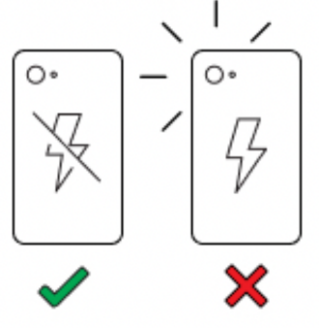 |
| 6 | **Device orientation**: The position of the mobile phone should be kept parallel to the light box | 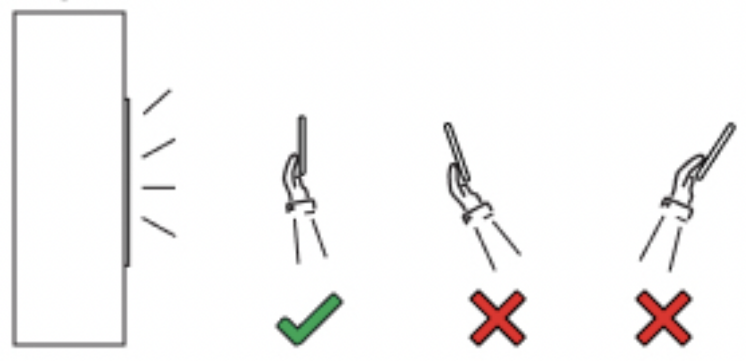 |
| 7 | **Focus:** The focus point of the mobile phone camera should be trained on the darker area of the film. | 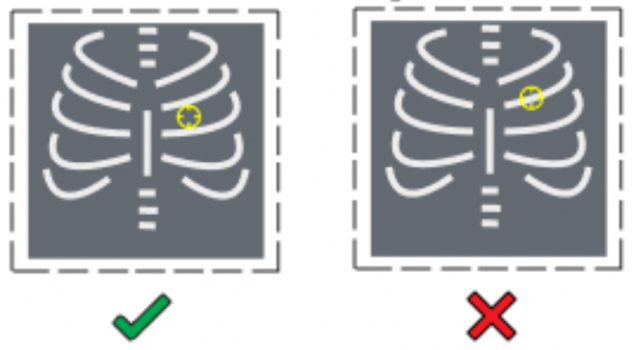 |
| 8 | **Visibility:** The apex and base of the lungs should be visible during the capture. | 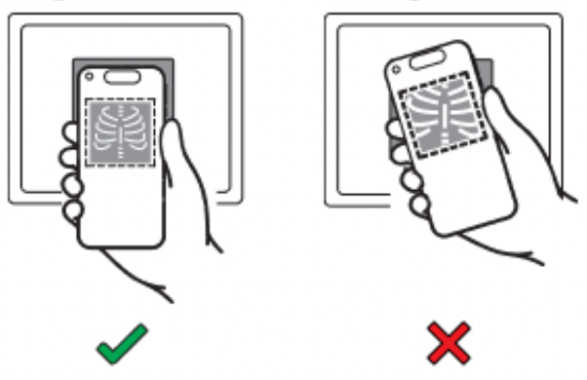 |
| 9 | **Cropping the area of interest:** Post image capture, the edges should be cropped before saving the image, as only the lung area should be in the frame | 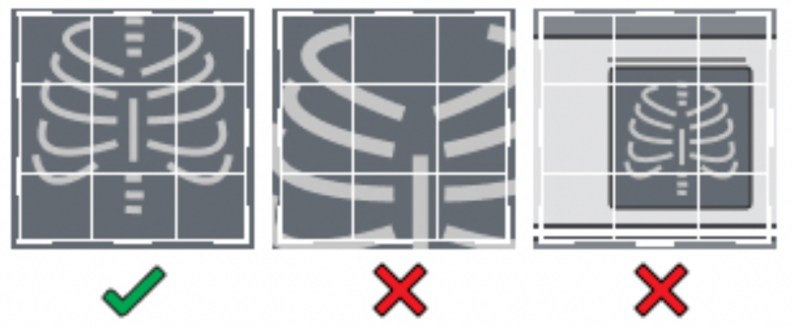 |
| 10 | **Image alignment:** The images should be checked while upload to ensure that they are not rotated or flipped. | 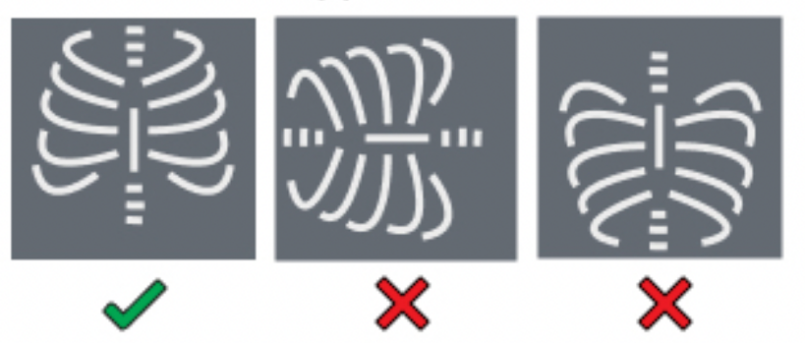 |
