# Supplementary material for: Evaluating the Usefulness of Artificial Intelligence-based Chest X-Ray Screening in Improving Tuberculosis Detection Among the High-Risk Tribal Population of Chhattisgarh, India: A Prospective Multi-Centre Study
Source: Open Forum Infect Dis. 2026 Jan 7;13(1):ofaf780. doi: 10.1093/ofid/ofaf780 (PMC12810203; doi:10.1093/ofid/ofaf780)

qure.ai

qXR – qTrack Orientation

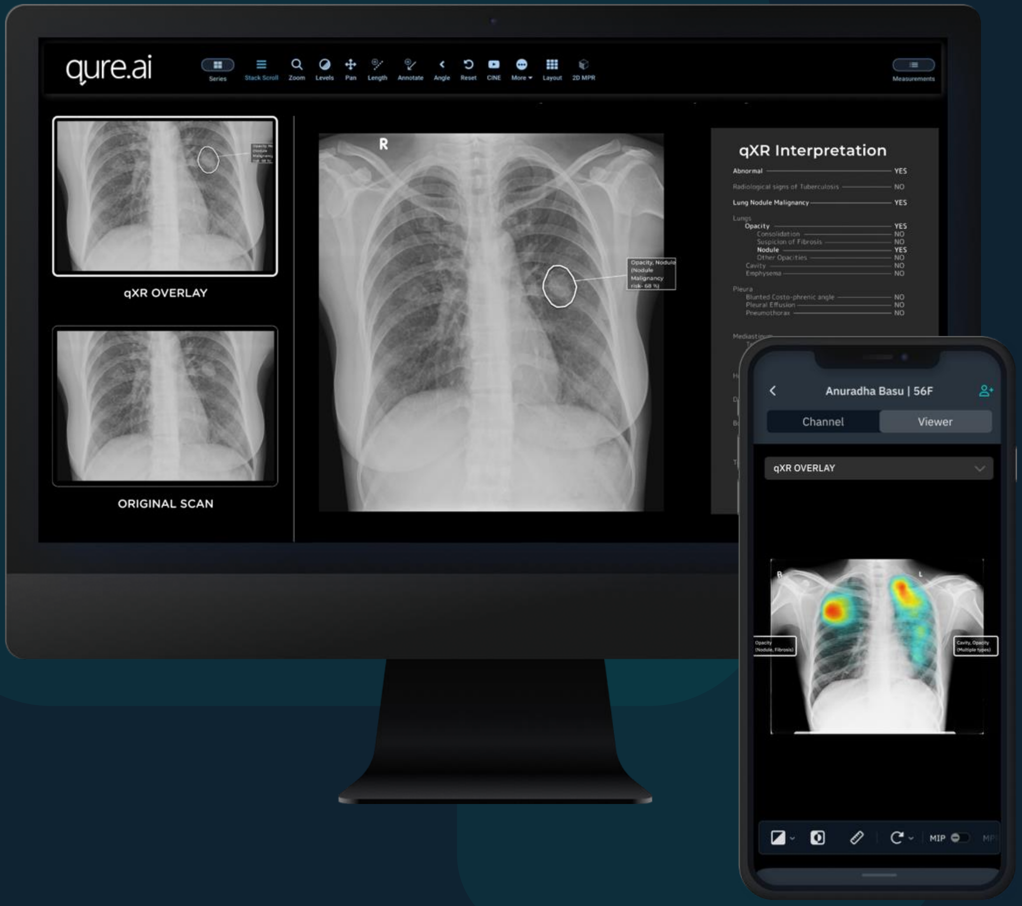

Product Overview – qXR

qure.ai

qXR: AI for Chest X-rays

510(k) cleared for Breathing tubes

Hardware & PACS Agnostic

CE Class II A Certified

WHO Endorsed

- Detects & localizes 30+ chest abnormalities inc. lung nodules
- Prioritizes worklist for abnormal chest x-rays
- Generates AI Secondary Capture of chest x-ray
- Pre-populates editable Radiologist report

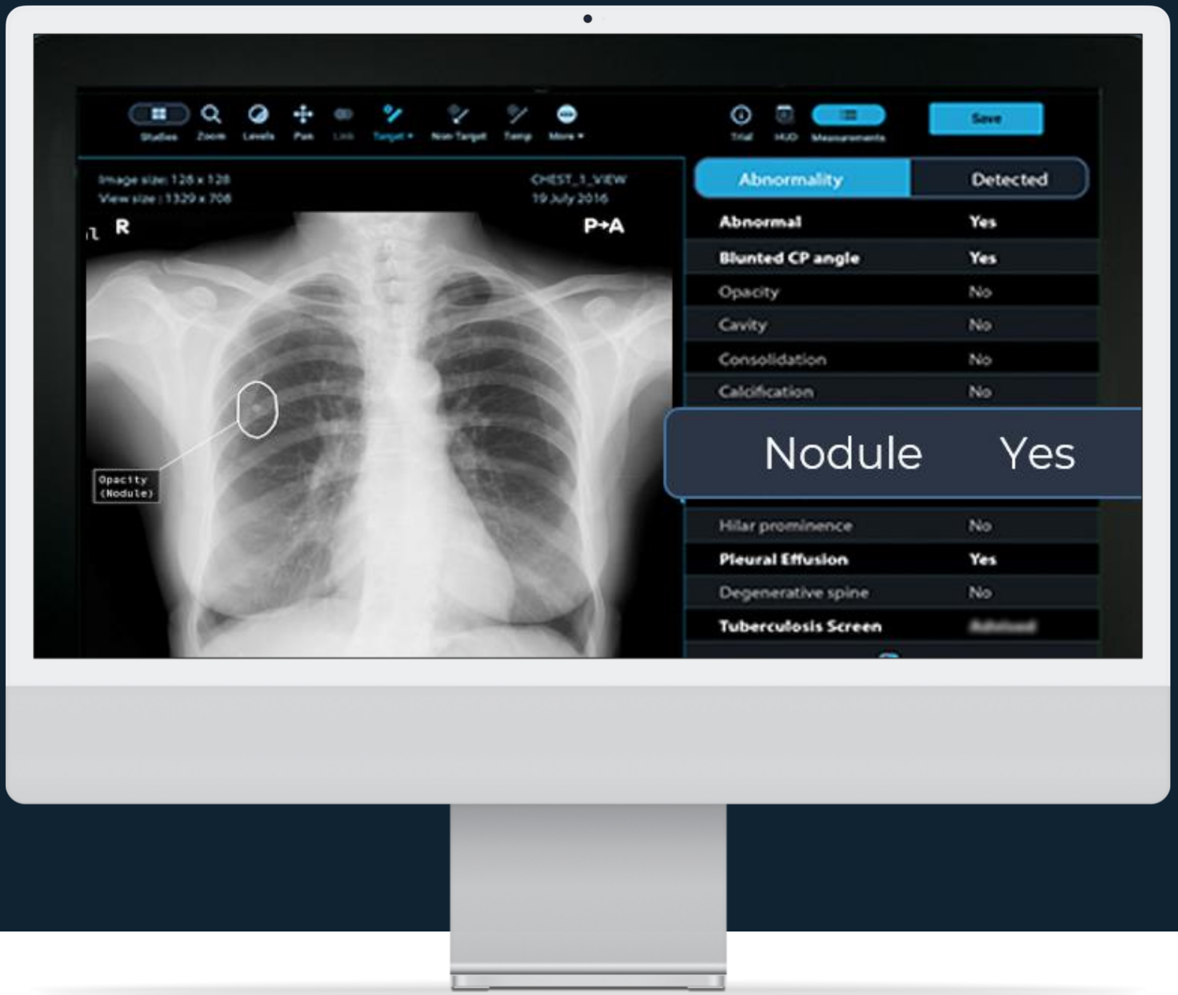

700+
sites, deployed across 50+ countries

4.5Mn+
Scans training dataset

7.3Mn+
Scans processed annually

Outputs

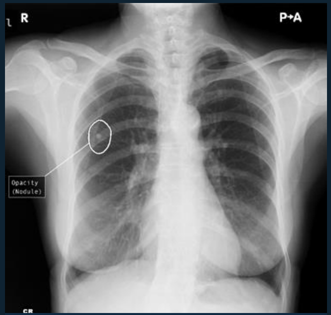

Nodule

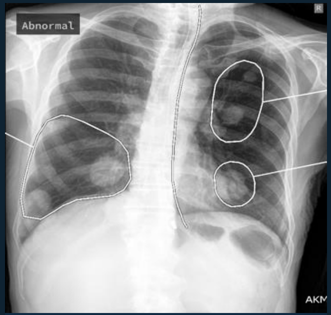

Opacities

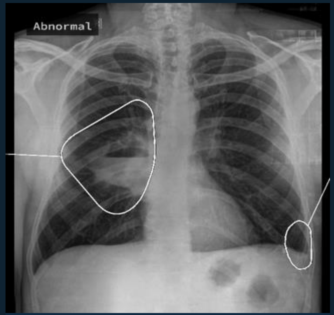

Cavity

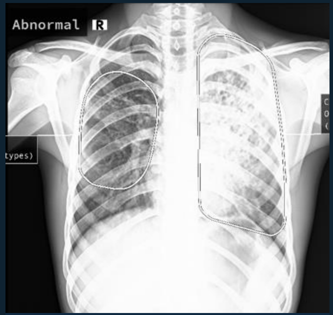

Tuberculosis

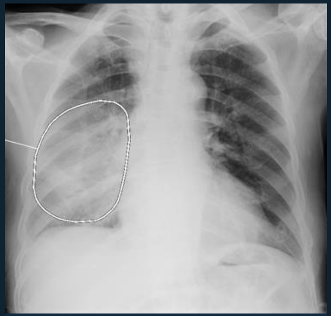
Consolidation

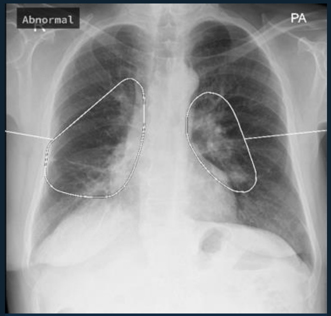

Fibrosis

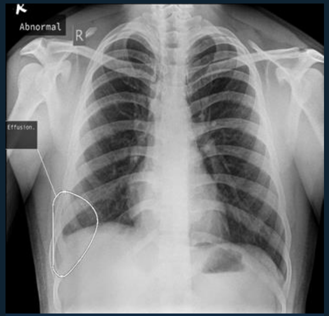

Blunted CP

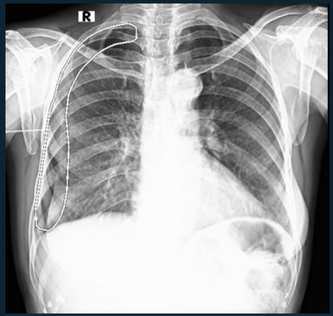
Pneumothorax

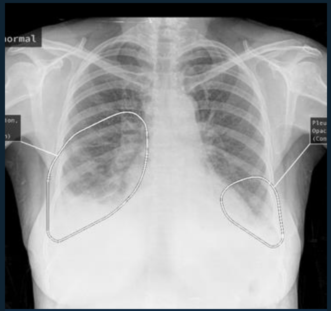
Pleural Effusion

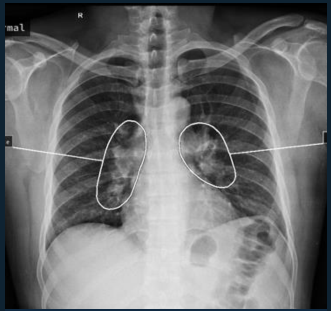
Hilar prominence

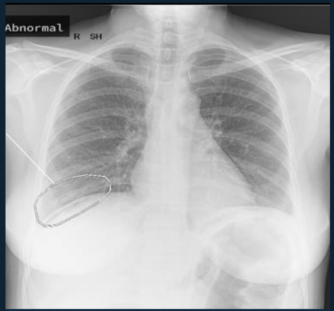

Free Air

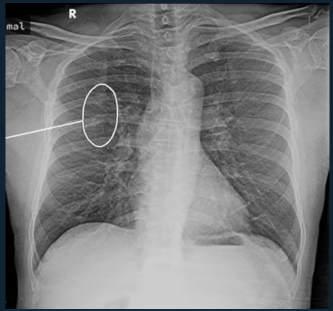

Rib fractures

Secondary Capture

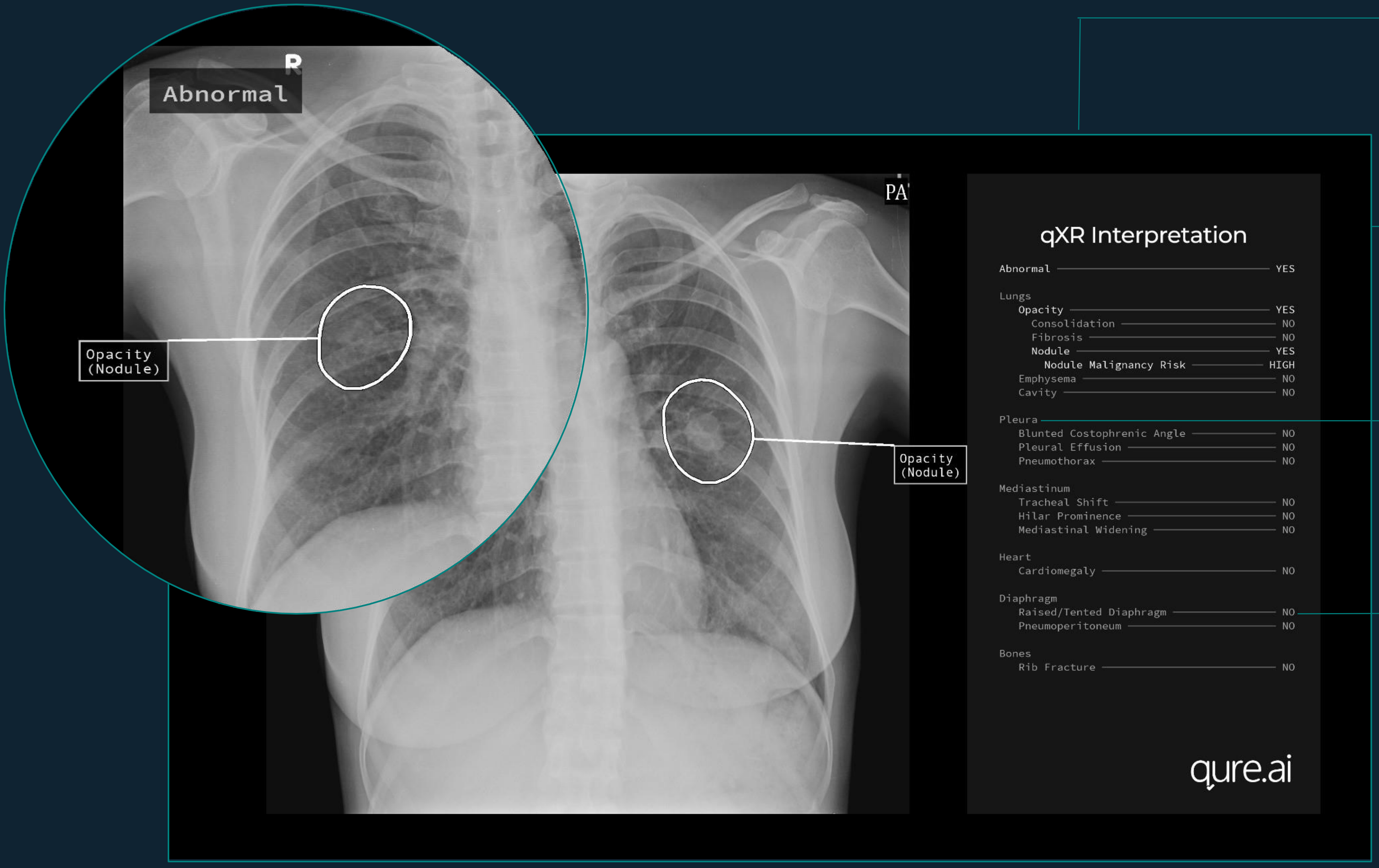

qXR annotates around the abnormality

Results available as an overlay on the SC

Abnormalities are indicated based on the region of chest

Simple YES/NO to depict whether abnormality is present or not

PDF Report

Location: cs_admin_nodule

CHEST X-RAY REPORT

THIS IS A SOFTWARE GENERATED REPORT AND IS NOT TO BE USED TO DETERMINE THE COURSE OF MEDICAL TREATMENT WITHOUT THE SIGNATURE OF A MEDICAL PRACTITIONER

Slip number:

Patient name: Jack Sparrow

Gender: Male

Age: NA

Patient code: NVB599448

Examination date: NA

Test code:

Referral Partner Name:

Referral Clinic:

FINDINGS (Click to edit the report)**:**

Opacity is observed in right lung and left mid, lower zones.
Inhomogeous Opacity, probable Consolidation is observed in right lung and left mid, lower zones.
Nodular Opacity observed in bilateral mid and lower zones.
Calcification is noted.

No blunting in CP angles is seen.

No significant findings observed in the heart.

Mediastinum is within normal limits.
Trachea is centrally located.

Radiologist Signature

DISCLAIMER

This is an auto-generated report by the software and cannot be used as the final report. Please treat this as a provisional report and correlate clinically. For any discrepancy, please consult a Radiologist/Chest Physician. The software has analysed for abnormalities in lung fields, mediastinum, pleura and for bone conditions. Any clinical diagnosis, misdiagnosis, or consequences to patient well-being and software performance changes or failures that may result directly or indirectly from integrating with the software will not be attributed to the software

qXR autogenerates an editable PDF report with all findings listed in full-text format

Disclaimer at the bottom of the report as it is a system generated output.

Provision for radiologists to sign at the end.

Digitizing and leveraging AI interpretation for analog X-rays using qTrack and qXR

qure.ai

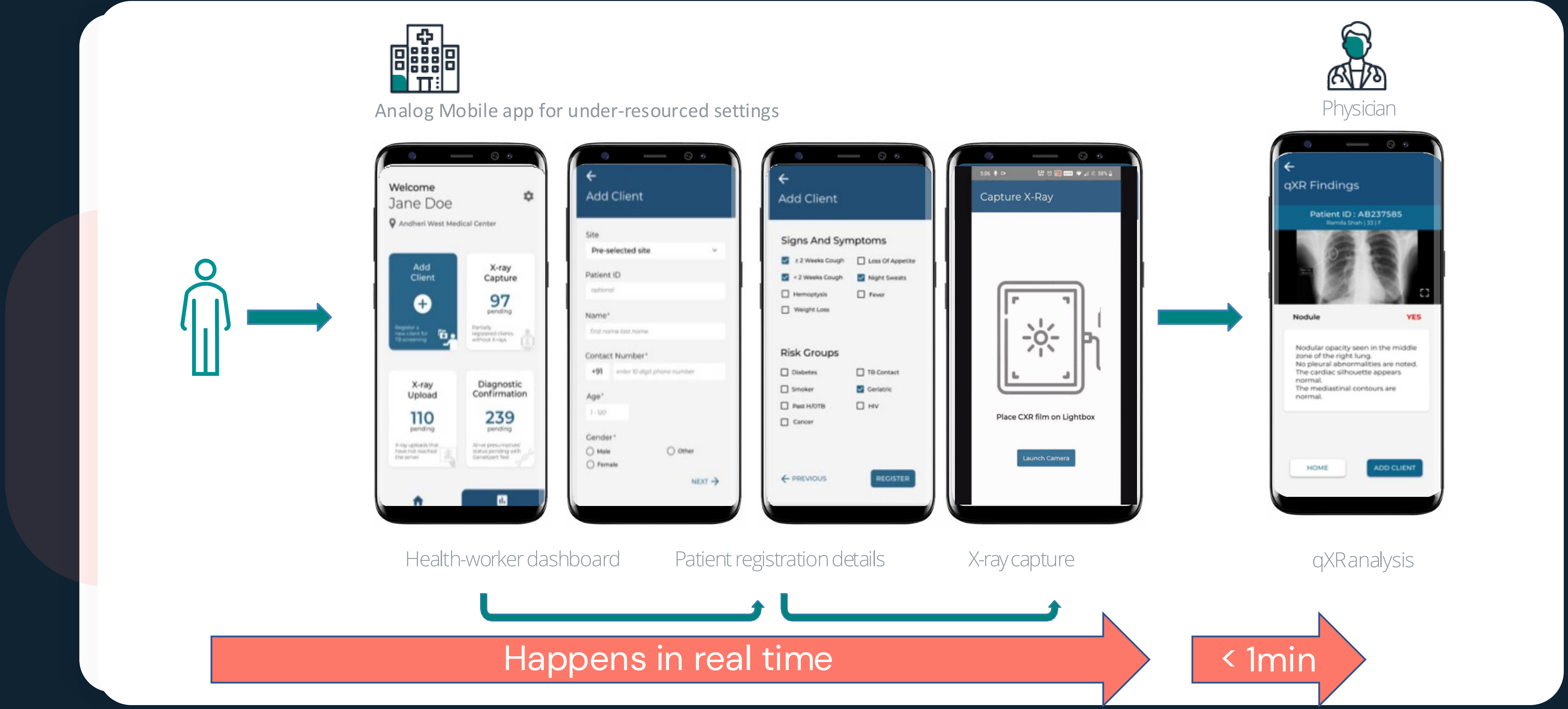

Installation

qure.ai

How to install qTrack

First, open your browser (Google chrome for android, Safari for iOS) and visit https://qtrack.qure.ai/ or scan the QR code.

For Android:

Click the menu at the top right-hand corner of your screen. Select the install option and the app will start downloading.

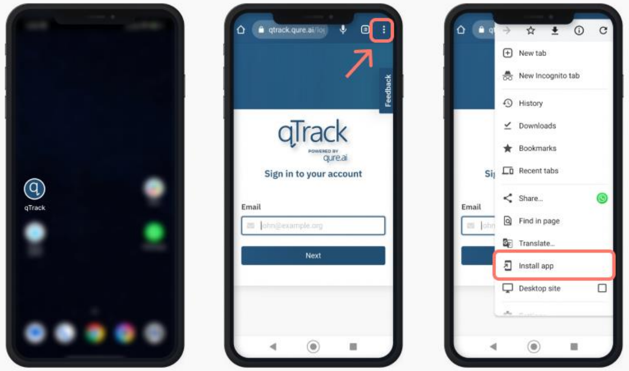

For iOS:

Open the menu at the bottom, click the option shown below and then “Add.” A bookmarked link will appear as a widget on your home screen.

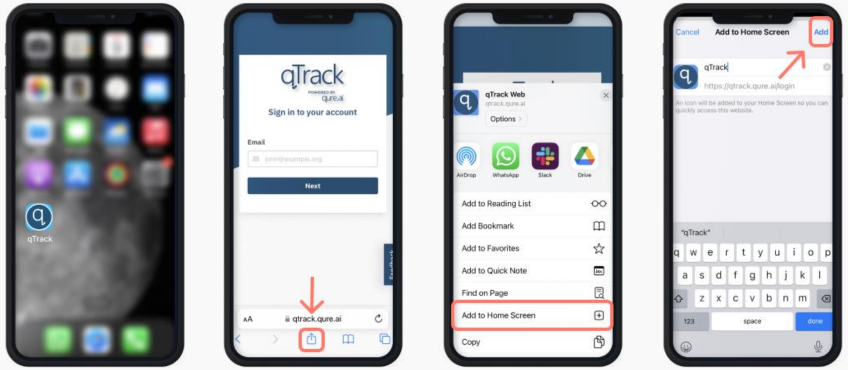

qTrack

On the frontlines

Physician

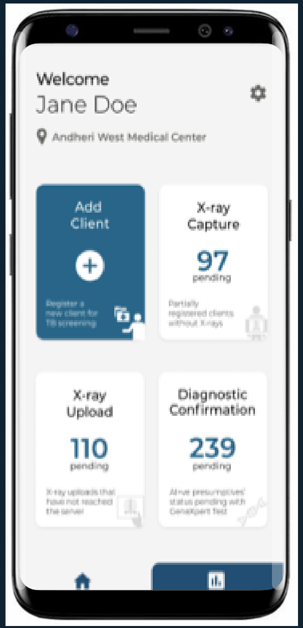

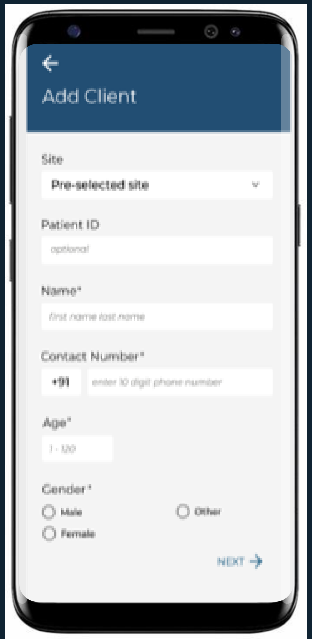

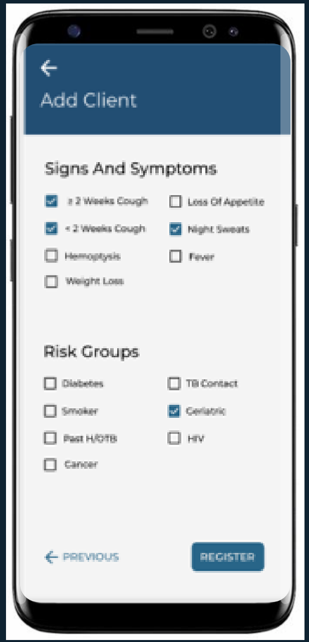

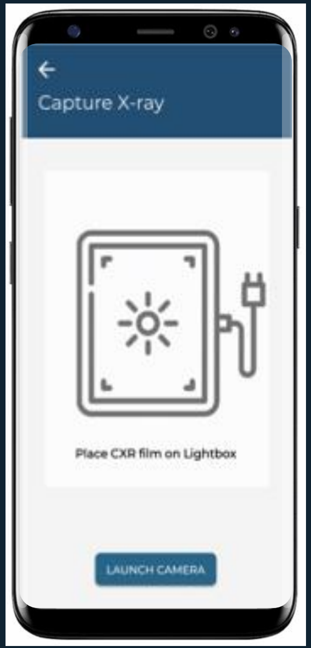

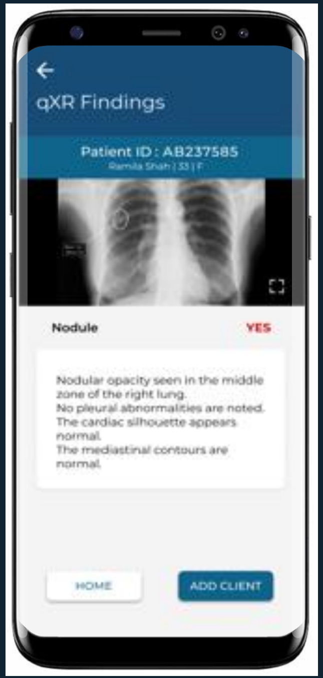

Health-worker dashboard

Patient registration details

X-ray capture

qXR analysis

Inclusion Criterion

qXR processes Chest X-Ray DICOM files that satisfy the following inclusion criteria:

- Body Part Examined: Chest
- View: PA / AP
- Patient Position: Erect / Supine
- Patient age should be > 6 years
- Image Resolution: Minimum of 1440x1440

Contraindications:

- Patient age is less than 06 years
- Lateral views
- Use on other anatomy or modality.

How to take X ray picture on qTrack app: https://www.youtube.com/watch?v=McCAFN7RR78

Demonstration – qTrack

qure.ai

Thank You!

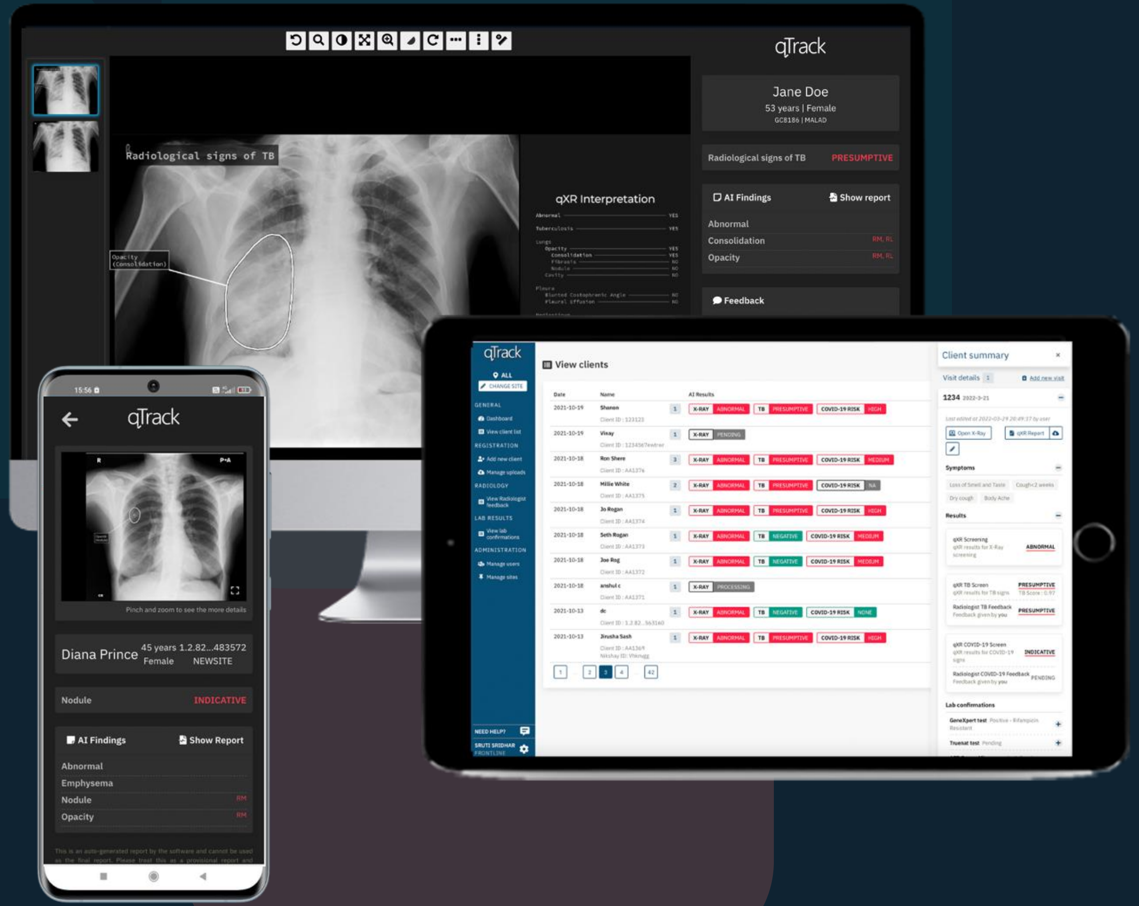

Supplement: ofaf780_Supplementary_Data [file ofaf780_supplementary_data.zip › Training Material_Supplementary Information.pdf]
