# Supplementary figures and images for: Evaluating the Usefulness of Artificial Intelligence-based Chest X-Ray Screening in Improving Tuberculosis Detection Among the High-Risk Tribal Population of Chhattisgarh, India: A Prospective Multi-Centre Study
Source: Open Forum Infect Dis. 2026 Jan 7;13(1):ofaf780. doi: 10.1093/ofid/ofaf780 (PMC12810203; doi:10.1093/ofid/ofaf780)

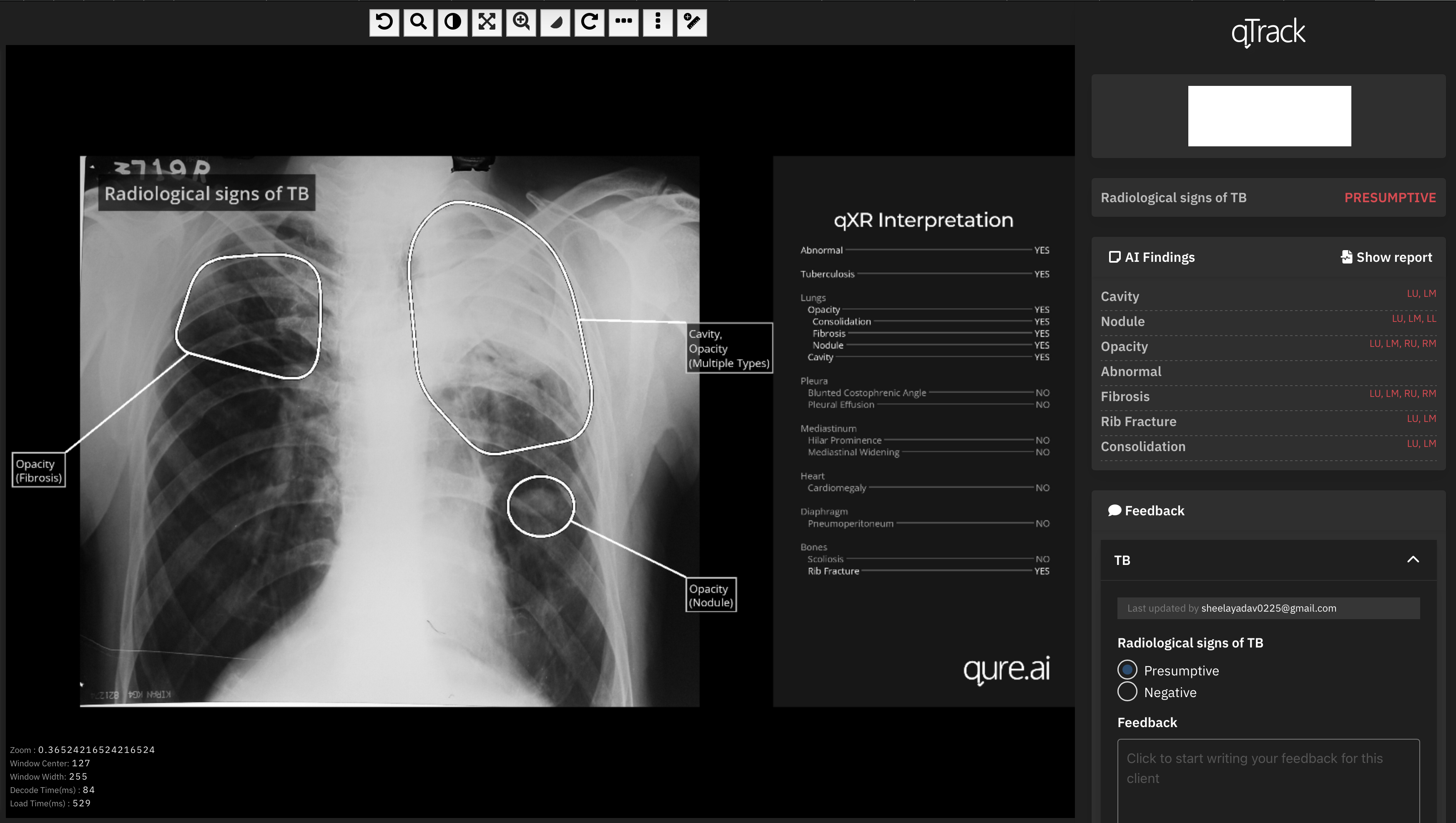

Supplement: ofaf780_Supplementary_Data [file ofaf780_supplementary_data.zip › S1.tiff]

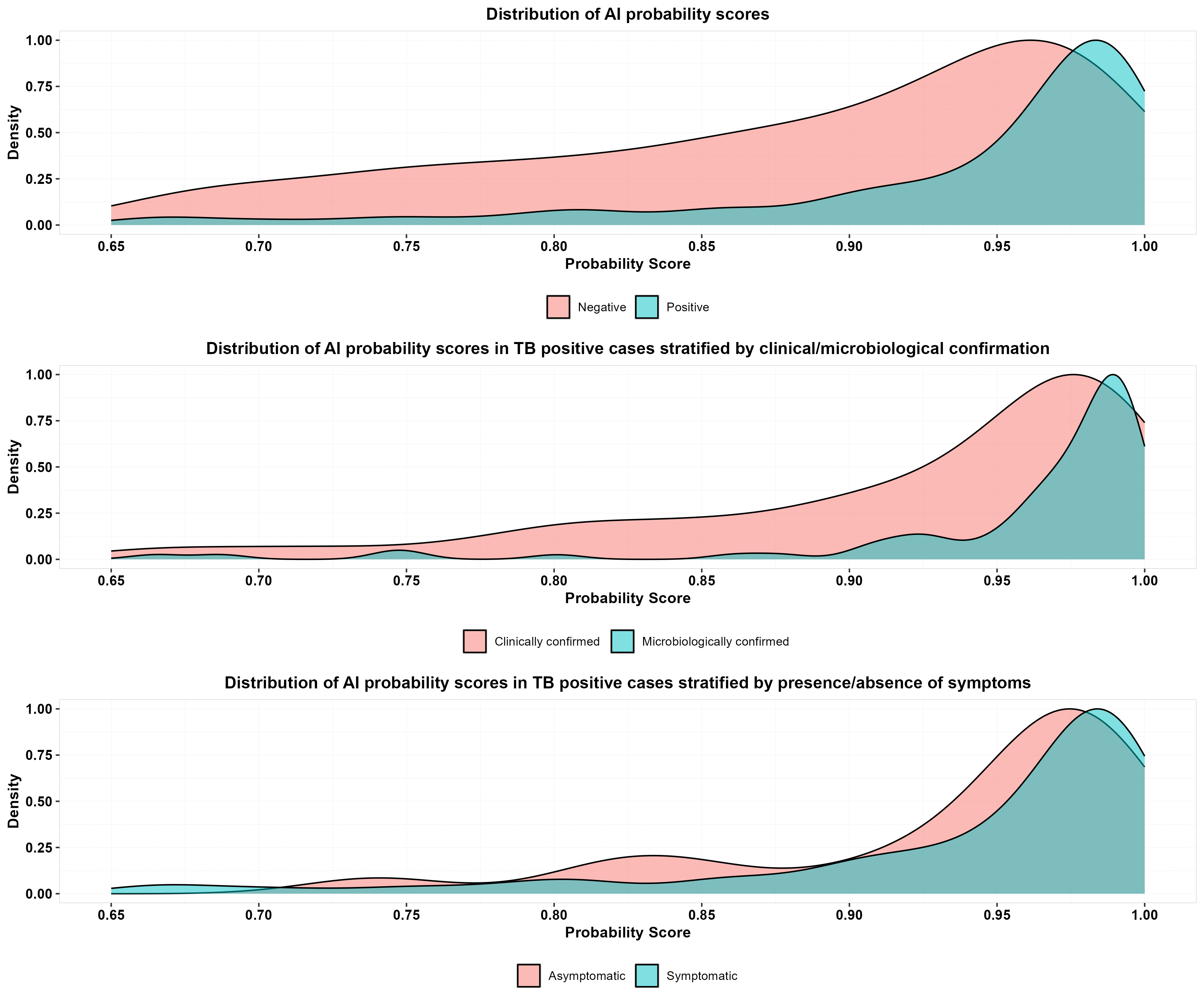

Supplement: ofaf780_Supplementary_Data [file ofaf780_supplementary_data.zip › S2.tiff]
